# The Expression Profile of MicroRNAs in Small and Large Abdominal Aortic Aneurysms

**DOI:** 10.1155/2019/8645840

**Published:** 2019-11-29

**Authors:** Václava Černá, Pavel Ostašov, Pavel Pitule, Jiří Moláček, Vladislav Třeška, Martin Pešta

**Affiliations:** ^1^Department of Biology, Faculty of Medicine in Pilsen, Charles University, Pilsen, Czech Republic; ^2^Biomedical Centre, Faculty of Medicine in Pilsen, Charles University, Pilsen, Czech Republic; ^3^Vascular Surgery Department, University Hospital in Pilsen and Faculty of Medicine in Pilsen, Charles University, Pilsen, Czech Republic

## Abstract

**Background:**

Abdominal aortic aneurysms (AAA) are relatively frequent in elderly population, and their ruptures are related with high mortality rate. There are no actually used laboratory markers predicting the AAA development, course, and rupture. MicroRNAs are small noncoding molecules involved in posttranscriptional gene expression regulation, influencing processes on cell and tissue levels, and are actually in focus due to their potential to become diagnostic or prognostic markers in various diseases.

**Methods:**

Tissue samples of AAA patients and healthy controls were collected, from which miRNA was isolated. Microarray including the complete panel of 2549 miRNAs was used to find expression miRNA profiles that were analysed in three subgroups: small (*N* = 10) and large (*N* = 6) aneurysms and healthy controls (*N* = 5). Fold changes between expression in aneurysms and normal tissue were calculated including corresponding *p* values, adjusted to multiple comparisons.

**Results:**

Six miRNAs were found to be significantly dysregulated in small aneurysms (miR-7158-5p, miR-658, miR-517-5p, miR-122-5p, miR-326, and miR-3180) and 162 in large aneurysms, in comparison with the healthy control. Ten miRNAs in large aneurysms with more than two-fold significant change in expression were identified: miR-23a-3p, miR-24-3p, miR-27a-3p, miR-27b-3p, miR-30d-5p, miR-193a-3p, miR-203a-3p, miR-365a-3p, miR-4291, and miR-3663-3p and all, but the last one was downregulated in aneurysmal walls.

**Conclusion:**

We confirmed some previously identified miRNAs (miR-23/27/24 family, miR-193a, and miR-30) as associated with AAA pathogenesis. We have found other, yet in AAA unidentified miRNAs (miR-203a, miR-3663, miR-365a, and miR-4291) for further analyses, to investigate more closely their possible role in pathogenesis of aneurysms. If their role in AAA development is proved significant in future, they can become potential markers or treatment targets.

## 1. Introduction

Abdominal aortic aneurysm (AAA), the enlargement of abdominal aorta to a diameter of at least 3 cm, is a common disease in the western part of the world, mainly in developed countries. The most common occurrence is in men over 65 years, where the prevalence is around 4–7% [1]. However, a significant percentage of the deaths caused by AAA rupture are also in women [2]. Due to high mortality rate when AAA ruptures (about 70–90%), it poses a serious health and public social problem in a lot of countries [3]. Although our understanding of this issue and its mechanisms is getting better, its complete aetiology is still unclear and we are still missing the crucial trigger in majority of AAAs. There are actually no prognostic laboratory markers used in clinical practice predicting the AAA behaviour. The identification of molecules involved in the deregulation of gene expression in the process of pathogenesis of AAA could be one option. Published data showed dysregulated expression of microRNAs in AAA tissue, and presumed the microRNA can play pivotal role in AAA development [4–6].

The positive family AAA history increases the risk of developing the same condition in relatives and indicates substantial portion of the genetic component. The heritability of AAA is over 0.7, and first-degree relatives of a patient with AAA have a 2-fold higher risk of developing an aneurysm as well [7]. Gene variants, found in genome-wide association studies (GWAS) associated with increased AAA risk, are usually not located in the coding regions and/or do not necessarily represent the causal ones. These gene variants may rather be in linkage disequilibrium with the causal alleles contributing to AAA formation, being located close to them [8]. Up to day, some risk loci on a few chromosomes were identified, but these explain only a small proportion of the heritability of AAA [7]. Positions of these genetic variants in noncoding regions suggest that they probably more often influence gene regulation than the protein structure [8]. The regulation of gene expression can be influenced in many ways including microRNAs (miRNAs).

miRNAs are short noncoding RNAs, first found and described at the very end of the last century, that are recently in focus of research due to their potential to become useful diagnostic and/or prognostic markers in a large variety of diseases. miRNAs are transcribed as long primary transcripts (pri-miRNAs) that are partly processed in the nucleus (pre-miRNAs) and finally in the cytoplasm. Mature miRNAs, about 20 nucleotides in length, associate with Argonaute family protein members and form RISCs (RNA-induced silencing complexes). RISCs interact with protein-coding mRNAs and inhibit their translation, or destabilize their molecules that are degraded and so decrease the levels of proteins coded by target mRNAs. On the other hand, in some cases miRNAs can also activate translation of target genes [9]. One miRNA can interact with many mRNAs, and, vice versa, one particular mRNA can be regulated by many different miRNAs. miRNAs can regulate gene expression either in the cell in which they were synthetized, or in other neighbouring or more distant cells, as they can be exported into circulation in the form of membrane-bound vesicles (exosomes and microvesicles), or in association with protein complexes [10].

A few thousands of miRNAs have been identified, over 2500 in the human genome. Some miRNAs, or significant changes in their levels, are associated with particular diseases, their stages, or acute events, and so became potential diagnostic or prognostic markers, for example, increased levels of miR-208a, miR-499, miR-1, and miR-133 in plasma are associated with myocardial damage [10]. The levels of miRNAs can be measured either in cells/tissue, or in circulation (serum, plasma, and blood), or other body fluids.

In this study, we aimed to evaluate the changes in 2533 miRNAs expression in small and large aneurysms compared to the normal vessel wall.

## 2. Materials and Methods

### 2.1. Patients

Our study included ten patients with small AAA (maximum diameter ≤ 5 cm), six patients with large AAA (>5 cm), and finally five “healthy controls” who were cadaveric organ donors. Only patients indicated for the AAA resection were included, either due to the size of AAA, or symptoms present. Patients with oncological diagnoses, autoimmune diseases, and mycotic or inflammatory aneurysms were excluded. Control aortic tissue samples were from the dead brain and heart-beating donors (without warm ischemia or any other tissue injury) and were collected at the same time as the kidneys were harvested and immediately frozen. All patients with AAA have signed an informed consent; cadaveric donors have been used based on the principle of presumed consent (approved by the local Ethical committee; decision from the 12^th^ of August 2014).

### 2.2. Tissue Samples

Tissue samples were collected within the aortic surgery. AAA samples during the open aneurysm resection, approximately 1 cm^3^ tissue sample from the anterior aneurysmal wall; control group samples from cadaveric donors, from the same region of aorta, were collected. Samples were immediately washed with physiological saline solution, cleaned from intraluminal thrombus, frozen with liquid nitrogen, and stored in the freezer (−80 degrees of centigrade).

### 2.3. miRNA Isolation

Sample aliquots (approximately 0.125 cm^3^) were frozen in liquid nitrogen and grinded to prepare homogeneous powder that was transferred into 1 ml of chilled TRI Reagent®RT (Molecular Research Center, Inc., Cincinnati, USA). Total RNA was isolated according to manufacturer's protocol (Manual Part Number: G4170-90011, Version 3.1.1, August 2015). Isolated RNA was dissolved in RNAse/DNAse-free water (Thermo Fisher Scientific Inc., Waltham, USA), and its concentration and purity were assessed spectrophotometrically using the Infinite M200 instrument (Tecan Trading AG, Männedorf, Switzerland). The RNA sample was stored at −80°C until further use.

### 2.4. miRNA Array

RNA samples were prepared for hybridization using miRNA Complete Labelling and Hybridization Kit (Agilent, Santa Clara, USA), following manufacturer's protocol, without optional spike-in control and purification of labelled RNA. Samples were randomized and hybridized for 20 hours on SurePrint G3 Unrestricted miRNA arrays with 8 × 60 K fields and Amadid 070156 (Agilent, Santa Clara, USA).

### 2.5. Data Analysis

After hybridization, microarrays were scanned using the Agilent G 4900DA scanner, and the resulting image was processed using Agilent Feature Extractor software version 11.5.1.1. Resulting data were processed using R version 3.5.1 with limma package version 3.36.3. The weights for imported probes showing saturation, nonuniformity, or significantly higher signal over background were set to zero. The background was then corrected using the normexp method with offset set to 50. The “cyclicloess” with “affy” method was used for normalization to allow comparison of samples between arrays. Probes annotated with systematic name of detected miRNA were considered as within array replicates and combined using “avereps function” from limma package version 3.36.3 to obtain the expression of particular miRNA. Fold changes between aneurysms and normal tissue were then calculated as well as corresponding *p* values. Benjamini & Hochberg method was used for adjustment of *p* values to multiple comparisons.

## 3. Results

### 3.1. Baseline Characteristics of Patients

Samples of 21 patients were analysed. Six were obtained from large aneurysms, ten from small aneurysms, and five from healthy controls. The baseline characteristics of patients are given in Table 1.

### 3.2. miRNAs Dysregulated in Small AAA

**Figure 1:**
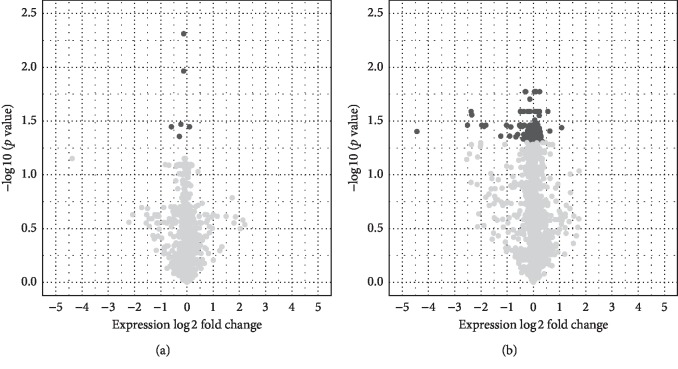
Comparison of miRNA expression in small (a) and large (b) aneurysms with healthy controls. Grey miRNAs (• dots) = nonsignificant; black miRNAs (• dots) = significant.

miRNAs expression in small and large aneurysm tissue was compared with healthy tissue. In small aneurysms, six miRNAs were detected with significantly different expression in comparison with the healthy aortic wall, including miR-7158-5p, miR-658, miR-517-5p, miR-122-5p, miR-326, and miR-3180 (Figure 1(a)). All of them were also significantly changed in large aneurysms. All but miR-326 were downregulated in the AAA tissue. In both large and small aneurysms the difference between the aneurysmal and healthy aortic wall was less than two-fold, and both had the same direction. Interestingly, fold changes in small and large aneurysms against healthy tissue were significantly correlated (Pearson's coefficient = 0.9521; *p* value = 0.0034).

### 3.3. miRNAs Dysregulated in Large AAA

In the large aneurysms, 162 differently expressed miRNAs were found (Figure 1(b); Table 2). Six of them were found to be also differently expressed in small aneurysms as mentioned in Section 3.2. Ten miRNAs with more than two-fold significant change in expression in large aneurysms were identified. These miRNAs included miR-23a-3p, miR-24-3p, miR-27a-3p, miR-27b-3p, miR-30d-5p, miR-193a-3p, miR-203a-3p, miR-365a-3p, miR-4291, and miR-3663-3p and all, but the last one was downregulated in aneurysmal walls.

## 4. Discussion

Based on miRNAs expression profiles, we have proved our assumption that some miRNAs are differently expressed in small and/or large AAA as compared to healthy tissue. In this analysis, we found six miRNAs dysregulated in small aneurysms in comparison with healthy controls (miR-7158-5p, miR-658, miR-517-5p, miR-122-5p, miR-3180 downregulated, and miR-326 upregulated). Twenty-seven times more miRNAs were found to be differentially expressed in large aneurysms, suggesting increasing miRNA levels dysregulation related to the disease progression. Despite higher number of small aneurysms than the large ones (10 vs. 6), we were unable to detect any other significantly changed miRNAs in comparison with healthy tissue, when correction for multiple comparison was applied. This suggests that small aneurysms are relatively close to the normal aortic wall regarding miRNAs expression.

As the number of differently expressed miRNAs is relatively high, we focus on miRNAs with more than two-fold significant change in expression (miR-23a-3p, miR-24-3p, miR-27a-3p, miR-27b-3p, miR-30d-5p, miR-193a-3p, miR-203a-3p, miR-365a-3p, miR-4291, and miR-3663-3p). Majority of data about these miRNAs come from studies focused on various types of cancer, where their roles in cell division, proliferation, migration, invasion, and apoptosis were identified. Some of them were analysed in cardiovascular diseases and miR-23/27/24 family and miR-193a also in AAAs.

### 4.1. MicroRNA-23/27/24 Family

miRNAs miR-27a, miR-23a, and miR-24-2 form an intergenic cluster on chromosome 19 (19p13.13), and miRNAs miR-23b, miR-27b, and miR-24-1 form an intronic cluster on chromosome 9 (9q22.32) [11]. Except for miR-23b, all were found to be downregulated in our samples of aneurysmal tissue. If no correction for multiple comparisons was applied, miR-23b-3p would be found significantly (*p*=0.004), more than two-fold, downregulated as well. All cluster members are highly conserved among vertebrates, and mature sequences of miR-24-1 and 24-2 are identical [11]. The miR-23/27/24 cluster members play a role in cell cycle control, proliferation, differentiation, and apoptosis. All of them are involved in regulation of angiogenesis; proangiogenic miR-23 regulates cardiomyocyte cell growth, promotes VSMCs proliferation, and inhibits VSMCs apoptosis by targeting the BCL2L11 (BIM) gene [12]; proangiogenic miR-27 is enriched in endothelial cells and highly vascularized tissues, inhibits endothelial cell growth targeting Rb, SEMA6A, and SEMA6D genes, negatively regulates MAPK and VEGFR2 signalling pathways [11, 13], and might contribute to plaque formation in atherosclerosis influencing MMP-13 (matrix metalloproteinase 13 or collagenase-3) expression in endothelial cells [13]; antiangiogenic miR-24 plays a pivotal role in endothelial cells apoptosis and angiogenesis, mediates contractile phenotype in vascular smooth muscle cells, and is upregulated in cardiac endothelial cells after cardiac ischemia and hypoxic condition [11]. To the validated miR-24 targets belong the endothelium-enriched transcription factor GATA2, the p21-activated kinase PAK4, the RAS p21 protein activator RASA1, and the histone-coding gene H2AFX that are involved in endothelial cells biology [11].

This miR-24 also affects smooth muscle cells proliferation, function, and apoptosis and inhibits proliferation, migration, and sprouting of human umbilical venous endothelial cells (HUVECs) [14]. Derived from the abovementioned facts, decreased levels of miR-23 attenuate its proangiogenic effect and downregulation of miR-27 and miR-24 results in the cell growth, angiogenesis, and loss of contractile phenotype. Maegdefessel et al. studied miR-24 in aneurysmal tissue and identified it as a key regulator of vascular inflammation and AAA pathology [15]. miR-24 was found to be colocalized with activated macrophages in aneurysmal aortic mouse tissue and visualized its expression in the aneurysm intimal-medial region. miR-24 downregulation was proinflammatory in macrophages [10]. The authors found miR-24 downregulated in both animal model and human AAA tissue (−1.9 ± 0.09-fold) versus control, and they found no significant differences between the small (52–67 mm; *n* = 12) and large AAA (69–115 mm, *n* = 10) but suggested a trend towards lower miR-24 expression with larger AAA. This downregulation is in good agreement with our findings in large aneurysms, as our subgroup of “large” AAA (diameter over 50 mm) involves both large and small Maegdefessel's groups. In addition, miR-27b was found to be downregulated in AAA in their study [15], which is in line with our results. Furthermore, levels of both miR-24 and miR-23b were significantly lower also in the intracranial aneurysmal tissue than in the healthy controls [16]. Our findings support the hypothesis that the downregulation of miRNA-23/27/24 family dysregulates angiogenesis, smooths muscle cells proliferation, function, and apoptosis, and together with their proinflammatory effect can contribute to the AAA development/progression.

### 4.2. miR-30d Family

The miRNA-30 family includes five miRNAs (miR-30a–e), and six mature miRNA molecules (miR-30a, miR-30b, miR-30c-1, miR-30c-2, miR-30d, and miR-30e) coded by six genes located on chromosome 1, 6, and 8, sharing a common seed sequence close to their 5′ ends [17]. The members of miRNA-30 family are involved in the process of angiogenesis via δ-like ligand 4 (DLL4) expression regulation [17].

We found miR-30b-3p and miR-30d-5p downregulated in large AAA tissue. If no correction for multiple comparisons was applied, miR-30a-3p, miR-30a-5p, miR-30b-5p, miR-30c-2-3p, and miR-30d-3p would be significantly downregulated as well (*p*=0.004). It is in line with published results of Spear et al., who found miR-30a-5p downregulated in the aneurysmal tissue of AAA patients in comparison with control tissues, and in addition, levels of circulating miR-30a-5p in plasma were also lower in AAA patients compared to patients PAD (periphery artery disease) and nonaneurysmal atherosclerosis (0.8-fold; *p*=0.04) [18].

In the cardiovascular system, the downregulation of miR-30 family contributes to endoplasmic reticulum (ER) stress and is associated with the upregulation of glucose-regulated protein 78 (GRP78) [19]. ER stress plays fundamental roles in the development and progression of various cardiovascular diseases such as ischemic heart diseases, heart failure, atherosclerosis, hypertension, and stroke [19]. Artificial knockdown of miR-30 triggered the phenotypic ER stress with significant GRP78/ATF6/CHOP/caspase-12 upregulations and cell death in rat cells [19]. In patients with acute coronary syndrome were found higher levels of circulating miR-30d-5p, in comparison with healthy controls (*p* < 0.001). The authors found this miRNA suitable as a diagnostic predictor of myocardial infarction [20]. In this context, the downregulation of miR-30 family members in our AAA samples could indicate an increased ER stress, dysregulation of endothelial cell growth, and increased cell death.

### 4.3. miR-193a and miR-365a

We found miR-193a-3p downregulated in AAA tissue samples. Without correction for multiple comparisons, all four analysed miRNAs-193 (miR-193a-3p, miR-193a-5p, miR-193b-3p, and miR-193b-5p) would be significantly downregulated. Gene coding miR-193a is on chromosome 17, while miR-193b is in cluster with miR-365a located on chromosome 16. Downregulation of miR-193a expression has been published by a few authors in both plasma samples [6, 21] and aneurysmal tissue of AAA/TAA patients [22, 23], and its levels were lower in plasma samples of patients with aneurysms before surgery than 5–7 days after surgery [21].

miR-193a-3p downregulates human endothelial cell proliferation and migration, and negatively regulates human circulating endothelial colony-forming cell (ECFC) vasculo/angiogenesis that contribute to vascular repair [19, 24].

All miR-365 analysed (miR-365a-3p, miR-365a-5p, and miR-365b-5p) were significantly downregulated in the aneurysmal tissue, miR-365a-3p more than two-fold.

miR-365a plays an important role in atherosclerosis, as it is proatherosclerotic and proapoptotic, and induces endothelial cell apoptosis [25]. miR-365 is upregulated in endothelial cells upon oxLDL (oxidized low-density lipoprotein) treatment, targets antiapoptotic protein Bcl-2, and promotes cell death [25]. Both miR-193a and miR-365a are involved in endothelial cell metabolism, and their dysregulation might therefore contribute to the aneurysmal pathology as well. Based on their published effects, their downregulation could increase proliferation, migration, and survival of endothelial cells.

### 4.4. miR-203a

Levels of miR-203a were lower in aneurysmal tissue. This miRNA is dysregulated in chronic inflammatory diseases that suggest its involvement in immune-mediated diseases [26]. miR-203 is highly expressed in skin (and keratinocytes) and oesophagus and overexpressed in patients with psoriasis in comparison with healthy controls [27]. One of its targets is suppressor of cytokine signalling-3 (SOCS-3), negative regulator of the STAT3 pathway that is activated by inflammatory cytokines. SOCS-3 is involved in cell growth, differentiation, and survival and in the regulation of immunity [27]. SOCS-3, a target of miR-203a, was shown to play a critical role in AAA development and dissection [28, 29]. The long-term suppression of SOCS-3 by increased levels of miR-203 may lead to stronger or prolonged inflammation [26]. As the inflammation is one of the key players in aneurysmal pathology [18], dysregulation of miRNAs influencing this process could contribute to the aneurysm formation.

### 4.5. miR-3663 and miR-4291

The expression of miR-3663 was upregulated, and the expression of miR-4291 was downregulated in the analysed aneurysmal tissue. Little is known about miR-3663. miR-3663-3p was identified as downregulated in tissue samples of patients with degenerative aortic stenosis, and miR-193-3p as overexpressed in comparison with heathy subjects [30]. This information is just opposite to our findings in aortic dilated tissue of AAA patients. We have not found any published specific functions associated with hsa-miR-4291.

The majority of miRNAs, we found dysregulated in the tissue of AAA patients, are potentially related to the aneurysmal pathology, being either involved in endothelial cell or smooth muscle cell metabolism and survival, or angiogenesis, or inflammation, or atherosclerosis (Table 3). Some of them (miR-24, miR-27b, and miR-193a) were identified in previously published literature as downregulated in aneurysmal tissue, or in plasma of patients with aortic aneurysms [15, 22, 23], what is in line with our findings. In addition to these three miRNAs, we identified miR-23a, miR-27a, miR-30d, miR-365a, miR-203a, and miR-4291 to be downregulated and miR-3663 upregulated.

In our analysis, a complete panel of 2549 miRNAs was tested. We found 6 miRNAs dysregulated in small and 162 miRNAs in large aneurysms in comparison with healthy controls. The number of samples in particular groups was limited, and therefore microarray results were not verified using qPCR. To compensate this main limitation of this study, the Benjamini and Hochberg method was used for adjustment of *p* values to multiple comparisons.

## 5. Conclusion

In our analysis, we tested the complete panel of 2549 miRNAs and we confirmed some previously identified miRNAs (miR-23/27/24 family, miR-193a, and miR-30) as associated with AAA pathogenesis. In addition, we identified other potential miRNA candidates not yet found dysregulated in AAA tissue (miR-203a, miR-3663, miR-365a, and miR-4291) for further analyses, to investigate more closely their possible role in pathogenesis of aneurysms. If their role in AAA development is proved significant in future, they can become potential markers or treatment targets.

## Figures and Tables

**Table 1:** Baseline characteristics of patients (number of patients and percentage in subgroups).

	Small aneurysms	Large aneurysms	Control
Number of patients	10	6	5
Age (years); mean/range	63 (54–75)	75 (63–83)	52 (37–63)
Man	9 (90)	4 (67)	4 (80)
Smoking	9 (90)	5 (83)	3 (60)
Arterial hypertension	7 (70)	5 (83)	2 (50)
Coronary artery disease	4 (40)	5 (83)	0 (0)
Peripheral arterial disease	5 (50)	1 (17)	0 (0)
Diabetes mellitus	2 (20)	1 (17)	0 (0)
Hyperlipidaemia	6 (60)	3 (50)	0 (0)
Chronic obstructive pulmonary disease	4 (40)	1 (17)	0 (0)

**Table 2:** List of 162 differently expressed miRNAs in large aneurysms, in comparison with healthy controls (*p* < 0.05).

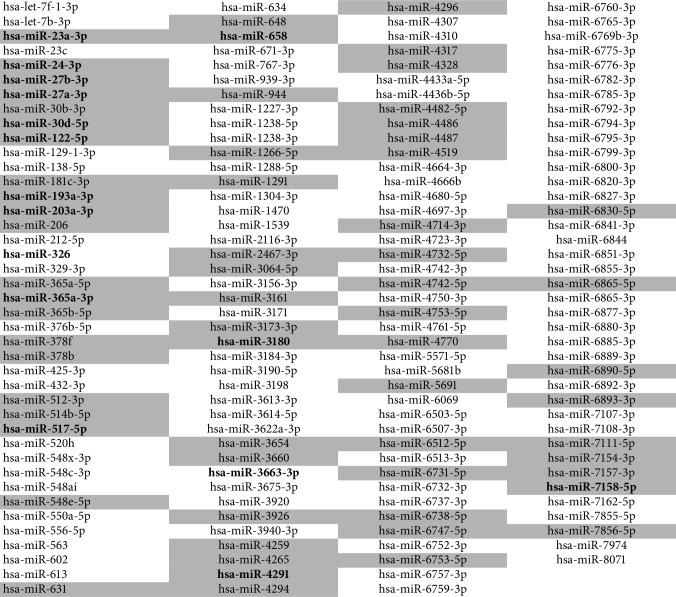

miRNAs in grey cells were downregulated and in white cells upregulated in the aneurysmal tissue; miRNAs bold and underlined were dysregulated in both, large and small aneurysms; miRNAs in bold italics are those with more than two-fold change in large aneurysms.

**Table 3:** miRNAs with more than two-fold significant change in expression in large aneurysm tissue, vascular biology processes in which they are involved, and their target genes.

miRNA	Process/function	Target genes	Reference no
miR-23	Angiogenesis (proangiogenic)	E2F1	[11]
	VSMCs proliferation		[12]
	Apoptosis (antiapoptotic)	BCL2L11 (BIM)	[12]
miR-24	Angiogenesis (antiangiogenic)	GATA2	[11]
	Apoptosis	GATA2, PAK4, BIM	[11]
	Mediator of contractile phenotype in VSMCs	Trb3	[11]
	Inhibits proliferation, migration and sprouting		[14]
	Vascular inflammation and AAA pathology		[10, 15]
miR-27	Angiogenesis (proangiogenic)	SEMA6A, SEMA6D, SPROUTY2, TSP-1, MMP-13	[11, 13]
	Endothelial cell growth inhibition	Rb	[11]
	Inflammation	MMP-13, VEGF, TSG-1	[13]
	Apoptosis	FADD	[13]
	Atherosclerosis	G2A	[13]
miR-30	Angiogenesis	DLL4	[17]
	Endoplasmic reticulum (ER) stress; cell death	GRP78, ATF6, CHOP, CASP12	[19]
miR-193	Angiogenesis (antiangiogenic)	HMGB1	[24]
miR-203	Inflammation	SOCS-3	[28, 29]
miR-365	Apoptosis	BCL2	[25]
	Atherosclerosis	BCL2	[25]
miR-3663	Downregulated in degenerative aortic stenosis	NA	[30]
miR-4291	NA	NA	—

ATF6: activating transcription factor 6; BCL2L11 (BIM): Bcl-2; CASP12: caspase-12; DLL4: δ-like ligand 4; E2F1: E2F1 transcription factor; FADD: Fas-associated protein with death domain; G2A: G protein-coupled receptor 132; GRP78: glucose-regulated protein 78; HMGB1: High mobility group box-1; CHOP: CCAAT-enhancer-binding protein homologous protein; MMP-13: matrix metalloproteinase 13; SEMA6A: semaphorin 6a; SEMA6D: semaphorin 6d; SPROUTY2: sprouty homolog 2; Trb3: tribbles-like protein-3; TSG-1: thrombospondin-1; TSP-1thrombospondin-1; VEGF: vascular endothelial growth factor; VSMCs: vascular smooth muscle cells.

## Data Availability

The data used to support the findings of this study are available from the corresponding author upon request.

## Abbreviations

AA: Aortic aneurysm
AAA: Abdominal aortic aneurysm
ATF6: Activating transcription factor 6
CHOP: CCAAT-enhancer-binding protein homologous protein
GRP78: Glucose-regulated protein 78
HUVEC: Human umbilical venous endothelial cells
IA: Intracranial aneurysm
RISC: RNA-induced silencing complex
TAA: Thoracic aortic aneurysm.
